# Targeted tibio-peroneal re-entry during subintimal revascularization using the Outback® catheter

**DOI:** 10.1186/s42155-021-00206-w

**Published:** 2021-01-28

**Authors:** K. Rippel, H. Ruhnke, B. Jehs, J. Decker, T. Kroencke, C. Scheurig-Muenkler

**Affiliations:** Department of Diagnostic and Interventional Radiology, University hospital Augsburg, Stenglinstr. 2, 86156 Augsburg, Germany

**Keywords:** Re-entry device, Targeted re-entry, Below-the-knee, Tibio-peroneal, SAFARI, Outback

## Abstract

**Background:**

Re-entry devices are used regularly in subintimal recanalization of chronic occlusions of the iliac and femoro-popliteal arteries and significantly contribute to the high success rate of these interventions. However, the use in tibio-peroneal arteries has only been described in few cases so far. The present work is a retrospective evaluation of the Outback® re-entry device for gaining targeted true lumen access at the level of the tibio-peroneal arteries.

**Methods:**

From 9/2017 until 10/2020 the Outback® catheter was used in case of failed spontaneous re-entry at the level of the tibio-peroneal arteries in 14 patients either instead of the usual retrograde approach via a pedal/distal-crural access (*n* = 11) or in combination with it (*n* = 3). Baseline demographic and clinical data, morphologic characteristics of the occlusions, procedural succedss, as well as the Society of Vascular Surgery (SVS) runoff score before and after revascularization were documented.

**Results:**

All patients (median age: 78 years; range: 66–93) suffered from peripheral artery occlusive disease Rutherford stage 4 to 6 with a median lesion length of 12 cm (range: 7–35). Technical and procedural success was achieved in all 14 patients. The mean re-entry accuracy was 0.25 cm (range: 0–0.8). The SVS runoff score improved from a median of 14.5 (interquartile range IQR: 10.8–16.4) to 7 (IQR: 6.3–7) (*p* < 0.01).

**Conclusions:**

The use of the Outback® catheter for targeted tibio-peroneal re-entry is associated with a high technical and procedural success rate and should be considered in case of otherwise failed ante- and retrograde recanalization.

## Introduction

As a result of demographic change and increasing life expectancy, the number of mainly older patients with clinically relevant peripheral arterial occlusive disease is increasing (Kreutzburg et al. 2020). The endovascular treatment of chronic total occlusions (CTO) of lower limb arteries causing claudication or critical limb ischemia is the treatment of choice nowadays and can be a great challenge (Hardman et al. 2014). Successful passage of the occlusion and subsequent angioplasty is not readily assured. In about 20% of all subintimal recanalizations of femoro-popliteal arteries spontaneous re-entry into the true lumen distally to the CTO fails and thus jeopardizes a successful revascularization (Shin et al. 2011). Re-entry catheters proved to be an efficient adjunct in case of failed spontaneous entry into the true lumen (Schneider et al. 2013). Below-the-knee recanalization of chronic occlusions in patients suffering from critical limb ischemia (Rutherford 4–6) can be even more challenging. The precise re-entry into the true vascular lumen and the avoidance of an unnecessarily long dissection is equally important here. The supporting retrograde probing via a pedal or distal-crural access is elegant and effective (El-Sayed et al. 2016; Bazan et al. 2014). However, retrograde probing may fail or be contraindicated. The aim of the present retrospective analysis is the technical evaluation of the Cordis Outback re-entry catheter (Cordis - a Cardinal Health Company, Santa Clara, CA, USA) for a targeted re-entry in cases where neither antegrade nor retrograde recanalization was reasonably possible. The Outback catheter is by far the best studied re-entry device available, however only about 10 cases of its use in tibio-peroneal arteries were published so far (Diamantopoulos et al. 2018; Aslam et al. 2013; Patrone et al. 2019; Tai and Lee 2015). The local ethics committee approved the retrospective analysis of anonymized patient data in the context of this study. The study was concordantly performed with the ethical standards of the 1946 Declaration of Helsinki and its later amendments.

## Materials and methods

### Study design and patient population

In this retrospective study all consecutive patients, who were treated within the 38 months period from September 2017 until October 2020 for CTO by using the Outback® catheter for targeted tibio-peroneal re-entry in subintimal recanalization, were included. Regarding demographic baseline data, age, gender, relevant co-morbidities and clinical stage of peripheral arterial occlusive disease according to Rutherford classification were documented (Hardman et al. 2014; Stoner et al. 2016). In addition, lesion characteristics, such as location and length of the occlusion, the degree of calcification documented as none, mild (< 25% circumference), moderate (25%–50%), or severe (> 50%) and the modified SVS (Society of Vascular Surgery) runoff score were recorded (Stoner et al. 2016). The latter ranges from 1 to 19, with higher values for more severe disease. The patency of the popliteal artery and the three arteries of the lower leg are assessed and any stenoses and occlusions are graduated. A score of 0 corresponds to a vessel with less than 20% stenosis, a score of 1 corresponds to a vessel with 21% to 49% stenosis, 2 corresponds to a vessel with 50% to 99% stenosis, 2.5 corresponds to a vessel that is occluded for less than half of its total length, and 3 corresponds to an occlusion of more than half of the vessel length. The value for the popliteal artery is multiplied by 3 and a value of 1 is added before all 4 vessel values are added. Particular attention was paid to all technical details of the revascularization, like the approach to recanalization, the location of the targeted re-entry as well as the accuracy of the Outback catheter in this respect and the subsequent angioplasty. The technical success of the re-entry manoeuvre itself as well as the overall procedural success defined as successful target vessel recanalization and improvement of wound perfusion were recorded including the achieved modified SVS runoff score. Complications were classified according to the Society of Interventional Radiology (SIR) Classification System for Complications by Outcome (Sacks et al. 2003).

### Procedure

All procedures were performed by one interventional radiologist (CSM) with more than 10 years of experience in vascular interventions and broad expertise in the use of the Outback® catheter, especially in iliac and femoro-popliteal recanalizations. All interventions were performed at a Philips Allura Xper FD 20 angiography system (Philips Healthcare, Best, The Netherlands). An antegrade approach via the ipsilateral common femoral artery was chosen for nearly all procedures. Only in one case the proximal superficial femoral artery had to be used as point of access due to obesity, punctured by ultrasound guidance. Access was gained using a Mini Access Kit (Merit Medical, South Jordan, UT, US) and after initial digital subtraction angiography of the leg arteries a 6 French sheath was introduced for the subsequent intervention. Under roadmap-guidance careful subintimal probing of the occluded vessel segment was performed using either a diagnostic catheter with a short angled tip (Cordis® Tempo® Vertebral Catheter, Cordis - a Cardinal Health Company, Santa Clara, CA, USA) in combination with an 0.035″ hydrophilic guidewire (Radiofocus® Guidewire M Standard Type, Terumo Corporation, Tokyo, Japan), or, in the majority of cases, a low-profile support catheter (0.018″ TrailBlazer™, Medtronic, Fridley, MN, USA) combined with an 0.014″ CTO wire (Hi-Torque Command ES guide wire, Abbott, Chicago, IL, USA). The spontaneous re-entry in the lower leg trifurcation area was unsuccessful in all of the presented cases. In this situation, a retrograde puncture and probing of the target vessel on the lower leg is usually attempted, unless wounds, necroses or extensive edema and inflammation in the pedal or crural access area prevent this. This applied to eight of the presented cases and was already considered during primary probing.

In two cases the distal posterior tibial artery was punctured in a retrograde fashion under ultrasound guidance using a Mini Access Kit. In another case the distal peroneal artery was punctured analogously under fluoroscopic guidance. After successful access, the occlusion was probed using an 0.014″ or 0.018″ Hi-Torque Command guide wire supported by an 0.018″ TrailBlazer catheter. This approach, also known as SAFARI-technique (Subintimal Arterial Flossing with Antegrade-Retrograde Intervention) is mostly successful, but in the three cases presented here no re-entry could be achieved in this way either (Spinosa et al. 2005). Therefore, a modified SAFARI-technique was used by introducing a 2.5 × 40 mm balloon catheter (Pacific Plus, Medtronic, Fridley, MN, USA) from the distal access point without prior placing of a sheath and dilated at the level of the tibio-peroneal trunc. The inflation of the balloon at the desired re-entry point with the following repeated probing with the wire did not lead to a successful re-entry. Hence, the Outback® re-entry device (Outback® LTD or Outback® Elite catheter, Cordis - a Cardinal Health company, Santa Clara, CA, USA) was introduced from the femoral access and placed parallel to the anew inflated balloon, which was then punctured directly. An 0.014″ Hi-Torque Command wire was guided into the balloon. When retracting the balloon, the wire could be pushed and pulled successfully further distal into the posterior tibial or peroneal artery, respectively, and finally out of the patient at the distal access point. Afterwards, a new balloon catheter was advanced from the femoral access by guiding it over the 0.014″ pull-through wire for subsequent angioplasty.

In almost all other cases retrograde pedal or distal-crural access was not possible due to above mentioned reasons. Only in one case retrograde access was avoided despite the absence of wounds in the puncture area, as retrograde probing seemed to be less promising due to an upstream occlusion of a femoro-popliteal bypass anastomosed to the distal P3 segment and severe calcification in the area of the trifurcation. Therefore, an 0.014″ Hi-Torque Command wire was advanced to the point of desired re-entry within the subintimal space. The Outback® catheter was then guided to that position. If a subintimal passage of the Outback® catheter was not possible due to friction, a prior balloon angioplasty with a 2 or 2.5 mm balloon was required. The correct positioning of the Outback® catheter was confirmed either by multiple angiographies in differently angled views or by using the plain fluoroscopy in case of heavily calcified vessels. In most cases, the Outback® needle was not fully deployed due to the smaller crural vessel diameter. In three cases, the shape of the needle was used to penetrate the anterior tibial artery in its proximal bend. The possible feeding of the wire with little to no friction in combination with roadmap guidance displaying the course of the target vessel properly was used to verify a successful re-entry. This was further confirmed by inserting a balloon catheter over the wire with the tip distal of the suspected re-entry point for a careful application of contrast agent. Afterwards, the balloon was used for an angioplasty of the entire subintimal tract and the re-entry point for an extended period. In case of residual stenosis due to dissection, recoil or heavy calcification self-expandable nitinol stents or stent-grafts (Astron Pulsar®, Biotronik, Berlin, Germany; sinus SuperFlex-418®, Optimed, Ettlingen, Germany; Supera®, Abbott, Chicago, IL, USA; GORE® VIABAHN®, W. L. Gore & Associates, Inc., Newark, DE, USA) were deployed to secure the result. In one patient a drug-coated balloon was used instead of placing a stent (IN.PACT® Admiral, Medtronic plc, Dublin, Ireland).

### Statistical analysis

According to their distribution, demographic and clinical baseline as well as follow-up data are given either as median with corresponding range or interquartile range (IQR, 25th – 75th percentile) or as mean with corresponding range. Statistical analysis was performed using R version 3.6 (https://www.r-project.org/). Wilcoxon’s one-sided signed-rank test for paired samples was used to compare the results of the modified SVS runoff score before and after revascularization. A *p*-value equal to or below 0.05 was considered statistically significant.

## Results

### Patient population and basic lesion characterization

A total number of 14 patients, eleven male and three female, were identified and included in this study. The median age was 78 years (range: 66–93), seven patients (50%) suffered from peripheral artery occlusive disease Rutherford stage 6, five (36%) of stage 5 and the remaining two (14%) of a Rutherford stage 4. The median lesion length amounted to 12 cm (range: 7–35) and included the popliteal artery in ten (71%) out of 14 cases. Target lesion calcification of seven (50%) patients was deemed mild, of four (29%) moderate and of the remaining three (21%) patients severe.

### Procedure

The Outback® was used either instead of the in such cases usual retrograde approach after pedal or distal-crural puncture of the target vessel or in three cases in combination with it. In two cases the Outback® catheter was employed in a second attempt after a previously aborted intervention. During these procedures distal subintimal recanalization failed and also led to a true lumen collapse of the target vessel. Therefore, a new attempt with a modified technique was started in a short interval of 3 days and 4 weeks, respectively, depending on the urgency of revascularization. In the remaining cases the re-entry device was used in the primary intervention. The experience that there are only limited possibilities for revascularisation of the lower leg and foot arteries in cases in which a retrograde access is not possible, influenced the further proceeding. Furthermore, it is important to consider that more aggressive subintimal manoeuvres could ruin the chance of revascularization by forming large dissections leading to a true lumen collapse of the small lower leg vessels. Therefore, probing was done carefully using mainly low-profile catheters and wires and the decision for a targeted re-entry using the Outback catheter was reached early. We identified four different technical scenarios, under which the presented cases can be summarized.

**Fig. 1:**
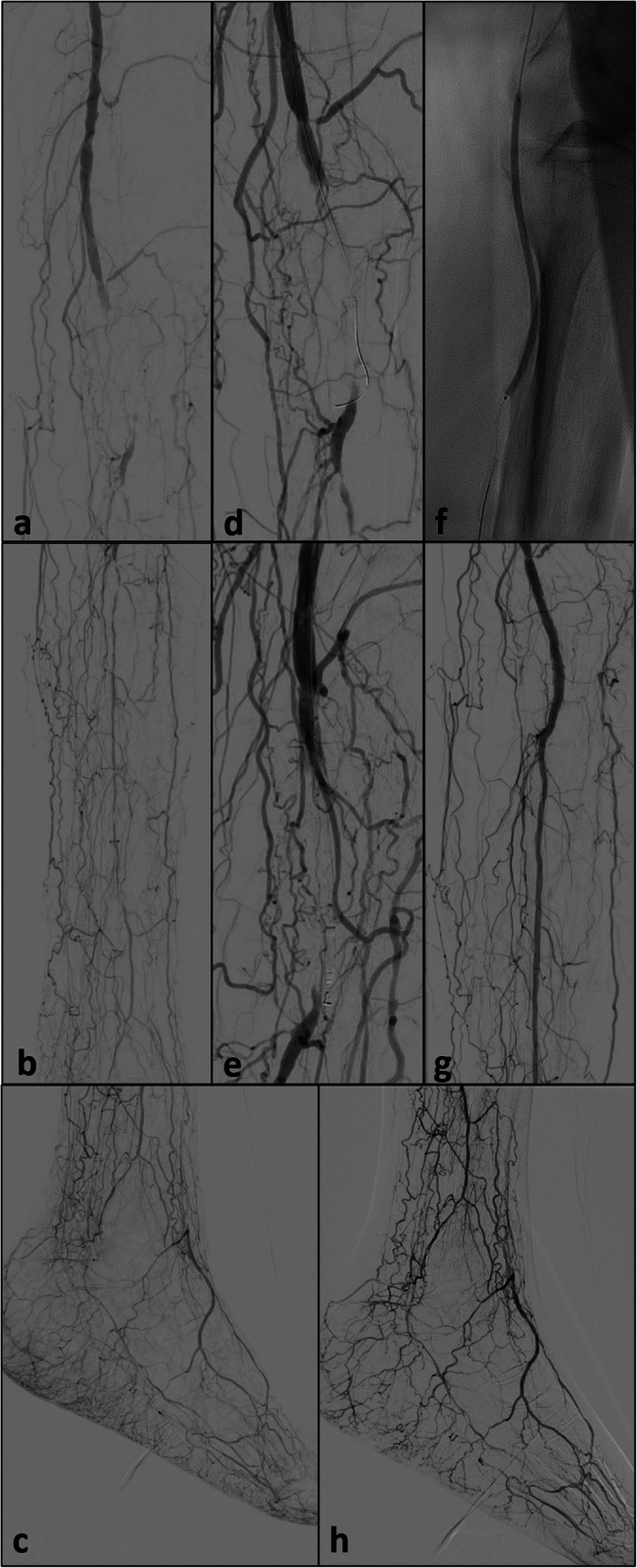
**a**, **b** and **c**: initial runoff prior to revascularization showing a chronic occlusion of the popliteal artery and besides collateral vessels a single vessel runoff via the considerably atherosclerotic peroneal artery; the tibio-peroneal trunc is in part preserved and ideal for a targeted re-entry in case of subintimal recanalization **d**: failed subintimal recanalization with the 0.014″ CTO wire within the subintimal space parallel to the true lumen but with a considerable large distance; a spontaneous re-entry was considered to be highly unlikely and targeted re-entry with the Outback® device chosen to complete the intervention; **e**: positioning of the Outback® catheter at the level of the tibio-peroneal trunc still a few millimeters too high and oriented to the wrong direction; after correct alignment the target lumen was accessed with a single needle deployment; **f**: balloon angioplasty of the distal popliteal artery, the tibio-peroneal trunc and the proximal peroneal artery after successful re-entry; **g** and **h**: final result after placement of a self-expandable stent (Biotronik Astron Pulsar®)

**Fig. 2:**
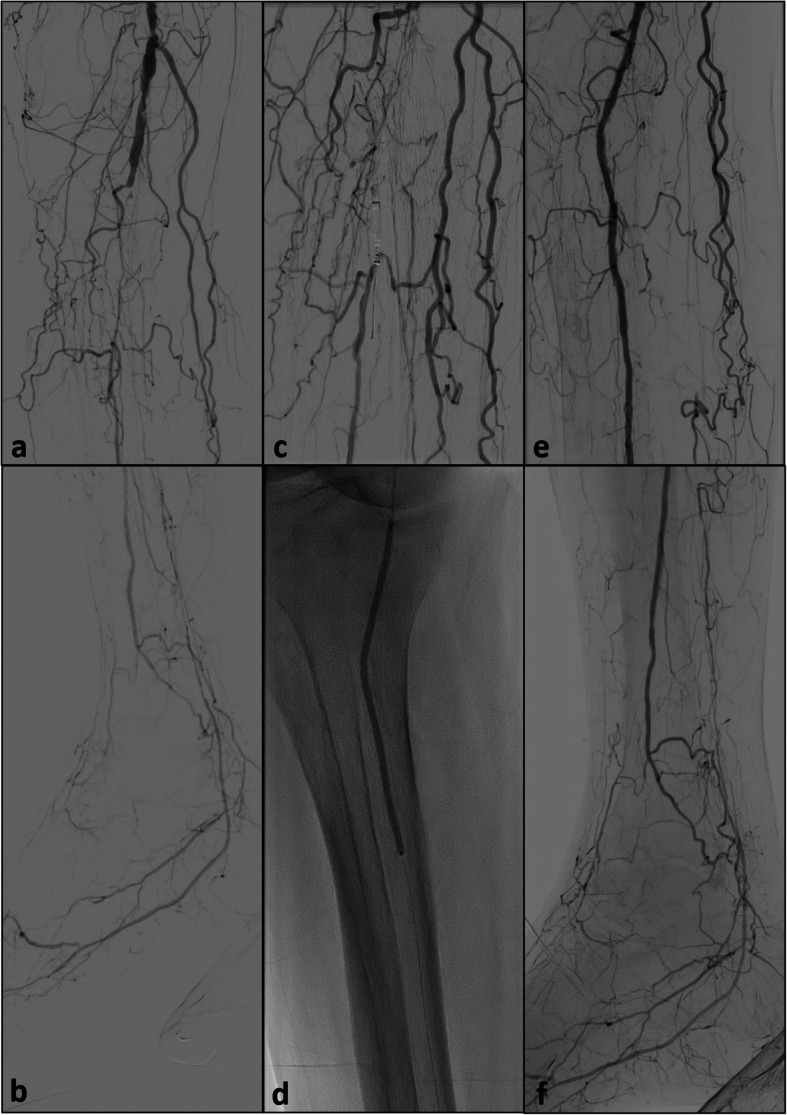
**a** and **b**: initial runoff prior to revascularization showing high grade stenoses of the proximal and middle segment and a chronic occlusion of the distal segment of the popliteal artery and the tibio-peroneal trunc with a single vessel runoff via the peroneal artery; careful subintimal recanalization using an 0.014″ CTO wire and a low-profile support catheter failed; **c**: positioning of the Outback® catheter at the level of the proximal peroneal artery with the 0.014″ wire still within the subintimal space parallel to the true vessel lumen and the device still a few millimeter too high for targeted puncture; the catheter was then advanced parallel to the perfused lumen of the peroneal artery; **d**: balloon angioplasty of the tibio-peroneal trunc and the proximal peroneal artery after successful re-entry; **e** and **f**: final result after successful revascularization

Scenario I (*n* = 5): Single vessel runoff via the peroneal artery with partial or complete occlusion of the tibio-peroneal trunc as well as considerable atherosclerosis of the further vessel course. Retrograde access was not possible due to massive edema, inflammation and/or extensive wounds. Antegrade subintimal probing failed (Figs. 1 and 2).

**Fig. 3:**
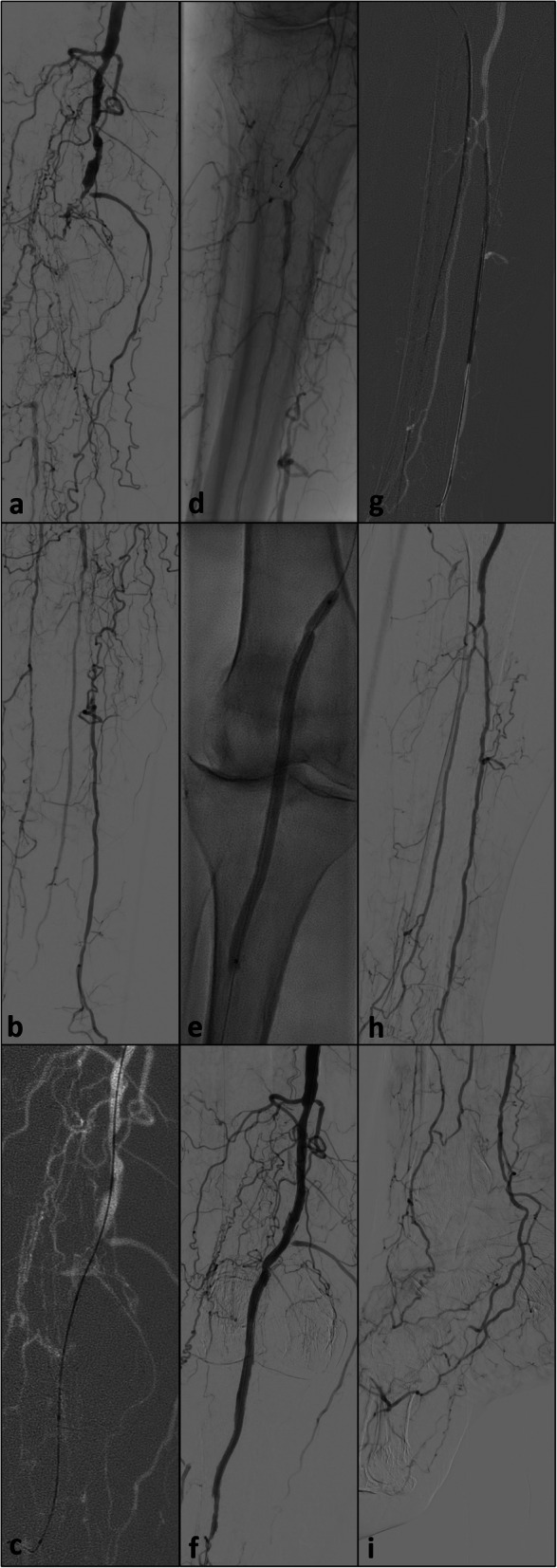
**a** and **b**: initial runoff prior to revascularization showing high grade stenoses of the proximal segment and a chronic occlusion of the middle and distal segment of the popliteal artery; preserved tibio-peroneal trunc and peroneal artery as well as anterior tibial artery, the latter, however, with a small caliber and impaired distal runoff to the foot; well preserved middle and distal segment of the posterior tibial artery as target vessel for restoring flow to the foot while maintaining perfusion of the peroneal artery; **c**: failed subintimal recanalization with the 0.014″ CTO wire and 0.018″ support catheter; **d**: targeted re-entry at the level of the tibio-peroneal trunc to enable further revascularization of the posterior tibial artery; **e** and **f**: angioplasty of the popliteal artery and placement of two Supera® stents; **g**: successful antegrade probing of the proximally occluded posterior tibial artery and subsequent angioplasty; **h** and **i**: final result after successful revascularization

Scenario II (*n* = 3): Occlusion of the popliteal artery and desired targeted re-entry at the level of the tibio-peroneal trunc with the aim of preserving both runoff vessels, the posterior tibial as well as the peroneal artery and not advancing a large dissection down to one of these vessels (Fig. 3).

**Fig. 4:**
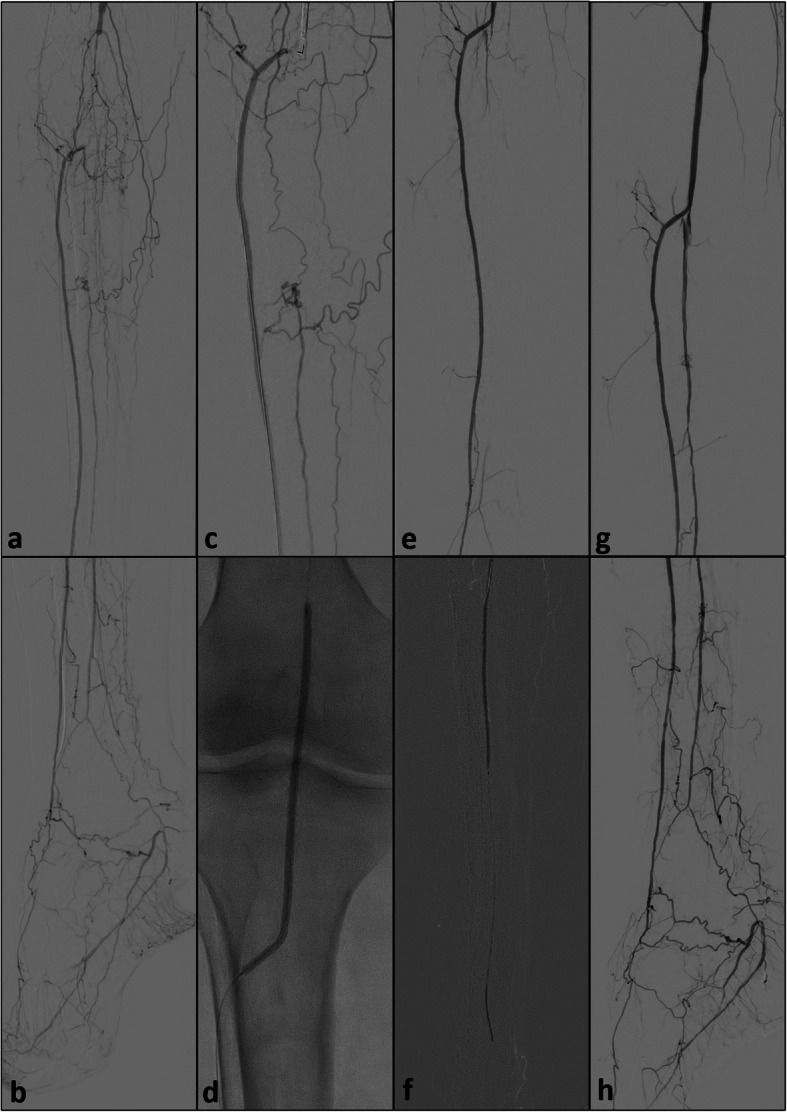
**a** and **b**: initial runoff prior to revascularization showing a chronic occlusion of the popliteal artery, the tibio-peroneal trunc and the proximal segment of the peroneal artery; well preserved anterior tibial artery as primary target vessel for recanalization; retrograde recanalization not possible dur to extensive wounds and inflammation; **c**: positioning of the Outback® catheter at the ostium of the anterior tibial artery with the 0.014″ wire already extending into the anterior tibial artery, the distal course being better visible due to movement artefacts; **d**: balloon angioplasty after successful re-entry and placement of a self-expandable stent (Optimed sinus SuperFlex-418®); **e**: result after angioplasty of the anterior tibial artery and now probing of the tibio-peroneal trunc and peroneal artery through the meshes of the placed stent; **f**: balloon angioplasty of the tibio-peroneal trunc and the proximal peroneal artery; **g** and **h**: final result with a restored two vessel runoff, however, with a dissection of the tibio-peroneal trunc not being flow limiting at the time of angiography, but certainly with a higher risk of re-occlusion

Scenario III (*n* = 3): Occluded popliteal inflow and single vessel runoff via the anterior tibial artery with preserved proximal vessel bend. Targeted puncture from the subintimal space of the popliteal artery into the course of the proximal anterior tibial artery (Fig. 4).

**Fig. 5:**
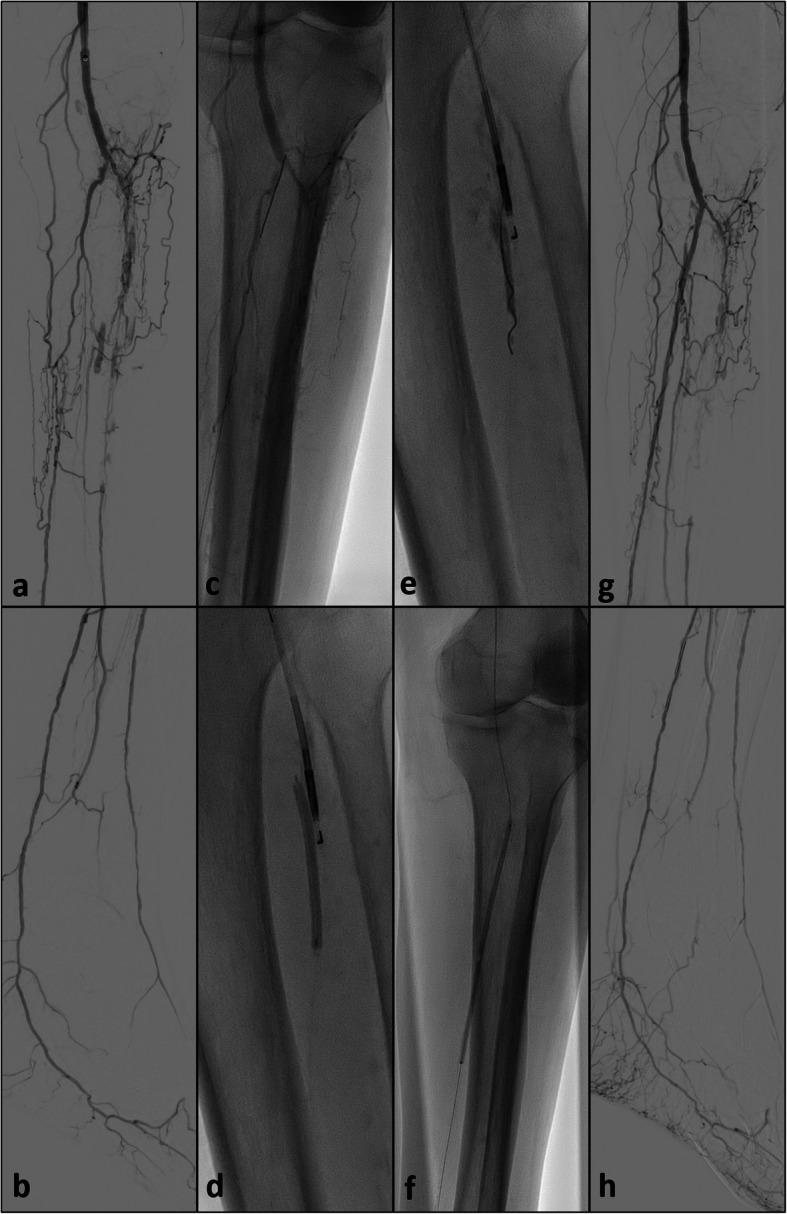
**a** and **b**: initial runoff prior to revascularization of the tibio-peroneal trunc and the posterior tibial artery; **c**: retrograde approach after puncture of the distal posterior tibial artery with failed spontaneous and balloon-assisted re-entry; **d** and **e**: positioning of the Outback® catheter from antegrade and placement of the angioplasty balloon from retrograde at the level of the tibio-peroneal trunc; hereafter, direct puncture of the balloon and feeding of the 0.014″ CTO wire into the bursted balloon; then retraction of the balloon and continuous feeding of the wire; **f**: balloon angioplasty of the tibio-peroneal trunc and the proximal posterior tibial artery after successful re-entry; **g** and **h**: final result after successful revascularization

Scenario IV (*n* = 3): Successful retrograde access of the posterior tibial or peroneal artery but failed access to the true lumen at the level of the tibio-peroneal trunc or the distal popliteal artery with subsequent application of the re-entry device assisted SAFARI technique at the level of the tibio-peroneal trunc (Fig. 5).

The device was employed at the level of the tibio-peroneal trunc in nine cases, three times at the level of the proximal anterior tibial artery and twice at the level of the peroneal artery. In all cases the subsequent angioplasty technique used to secure the point of re-entry and to restore blood flow consisted of plain balloon angioplasty. However, in one case an additional drug-eluting balloon was employed. Furthermore, in nine cases the additional placement of stents at the point of re-entry was deemed necessary, using a 4 mm sinus SuperFlex-418® in three cases, a 4 mm Astron Pulsar in three cases, a 4.5 mm Supera® in another two cases and a 5 mm VIABAHN® in one case. In four of these cases additional stents or stentgrafts were deployed during upstream vessel recanalization. Figure 4 demonstrates the procedural steps of the modified SAFARI-technique with the combined use of a distal-crural access and the antegrade advanced re-entry device.

### Evaluation of success

**Fig. 6:**
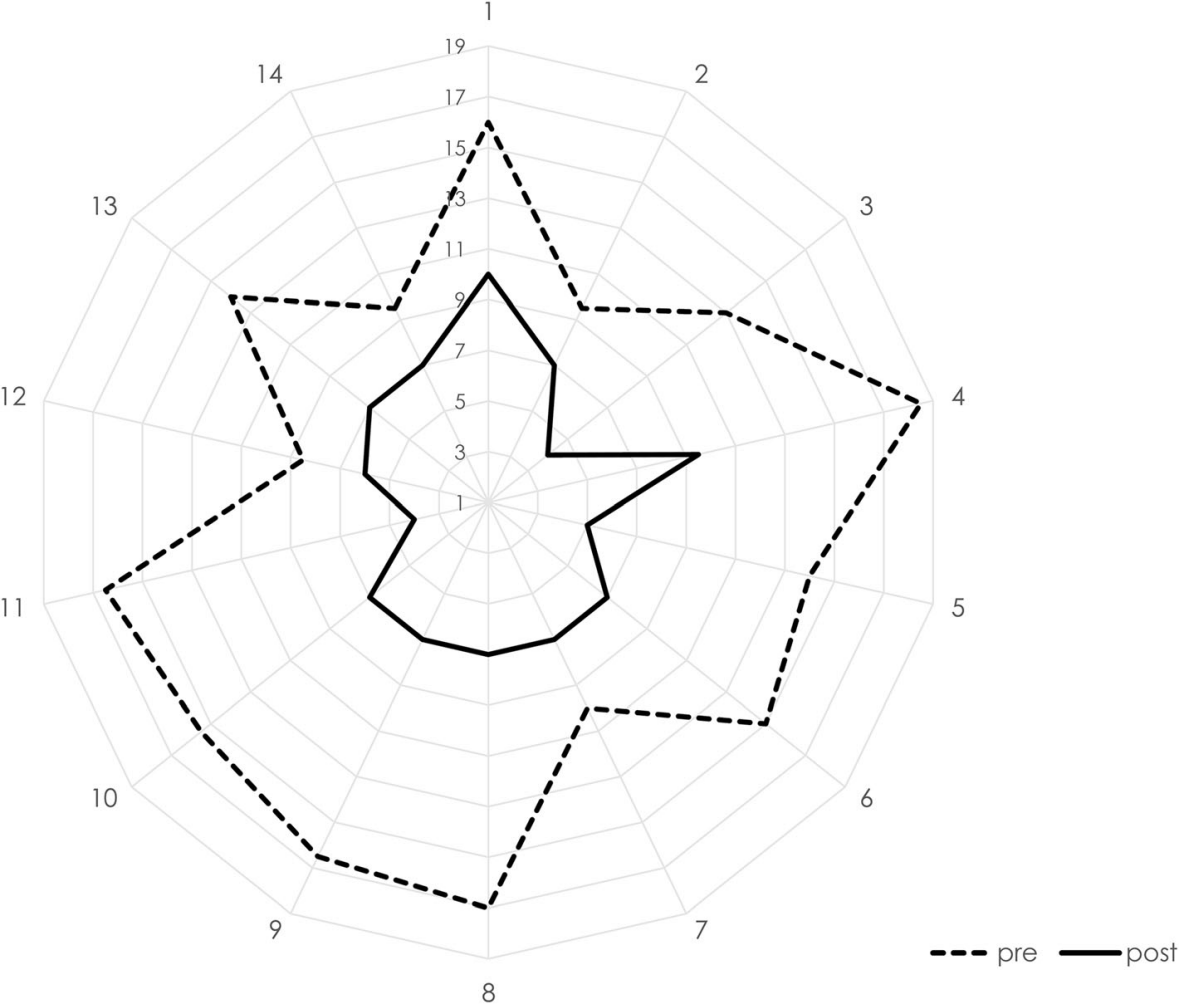
Individual improvements of the SVS (Society of Vascular Surgery) runoff score for each patient visualized on a radial plot with higher values indicating a more severe degree of stenoses and occlusions. Each spoke represents the score of one patient

Technical and procedural success was achieved in all 14 patients. Targeted re-entry was successfully achieved with a mean re-entry accuracy of as little as 0.25 cm (range: 0–0.8). The median SVS runoff score improved significantly from 14.5 (IQR: 10.8–16.4) points prior to revascularization to 7 (IQR: 6.3–7) points afterwards (*p* < 0.01). Figure 6 illustrates the individual improvements of the runoff score for each patient visually on a radial plot. For none of the presented cases a major complication as defined by the SIR classification was documented.

## Discussion

As of right now the Outback® re-entry device is by far the best scientifically investigated system, especially for its use in the aorto-iliac and femoro-popliteal region. As numerous studies have shown, the device has a high technical success rate of 87 to 100% when used for femoro-popliteal re-entry and of 91% when employed for an aorto-iliac re-entry (Etezadi et al. 2010; Gandini et al. 2013). Major complications were not described in these studies.

Besides these widely spread forms of use, the Outback® is also established for the fenestration of dissection-membranes during the treatment of aortic dissections (Wolfschmidt et al. 2015). Furthermore, there are some reports about the usage of the Outback® catheter in selected scenarios, e.g. in the retrograde recanalization of the superior mesenteric artery or for a retrograde recanalization of an occluded superficial femoral artery (Patrone et al. 2019; Ben Abdallah et al. 2020).

However, there are only few reports about the application of the Outback® re-entry device below the popliteal level. So far, ten cases have been published (Diamantopoulos et al. 2018; Aslam et al. 2013; Patrone et al. 2019; Tai and Lee 2015). Diamantopoulos et al. described the overall technical success rate of the infragenicular employment of the Outback® as 90%. However, it is not possible to differentiate between technical success rate of the Outback® catheter at the level of the distal popliteal artery and the tibio-peroneal arteries (Diamantopoulos et al. 2018). The technical success rate of all other published cases was 100% (Aslam et al. 2013; Patrone et al. 2019; Tai and Lee 2015).

In the retrospective evaluation at hand, we succeeded in confirming the high technical and procedural success rate, reaching 100% in the 14 presented cases with application of the device at the level of the tibio-peroneal arteries. We deliberately refrained from also including distal popliteal applications, as these, although being below the knee, to our experience are technically not different to applications in the above the knee femoro-popliteal arteries. The high success rate may in part be due to the broad experience of the interventionalist. But the system has a short learning curve and with a little experience in the femoro-popliteal area should also be reliably applicable to the lower leg arteries. However, it should by no means serve as a primary option but should be contemplated in cases in which puncture and retrograde probing are not possible. However, there are some technical issues that should be considered, when planning to apply this technique. In order not to jeopardize the possibility of applying the Outback® catheter, subintimal probing at the level of the targeted re-entry should be done carefully using low profile CTO wires and catheters. Large dissections may lead to true lumen collapse or simply confusing luminal relations impeding targeted puncture. In such situations a termination of the intervention and a second attempt a couple of days later should be considered. When applying the catheter, one also must be aware of the considerably smaller distances compared to the femoro-popliteal arteries. The needle should be extended carefully and usually not to its full length. It is also easier to accidentally puncture one of the accompanying veins instead of the artery, which should be ruled out by runoff angiography before angioplasty. Usually, it is enough to perform an angiographic run from the inguinal sheath to verify, whether the advanced wire projects onto the course of the target artery (see, for example, Figs. 3d and 4c). Alternatively, one may remove the Outback® device and advance the desired low-profile balloon into the target artery, retract the wire and perform a runoff visualization via the lumen of the balloon. A noteworthy weakness of the catheter system is its bulkiness. Since the device needs to be advanced far into the lower leg, this usually demands an ipsilateral antegrade access and automatically implies a high stress on the small crural vessels and the surrounding tissue and possibly a higher risk of damage. Despite these potential concerns, the system was applied in all cases similar to those in the previously published studies without any relevant complication, however, mainly in the upper third of the crural vessels close to the trifurcation. Applications more distally are possible, but may fail in cases with very thin, severely calcified or angulated vessel courses. This also has to be considered when planning to use the Outback® catheter as a bail-out-option. Diamantopoulos et al. also presented a case of a very distal and successful application of the Outback® catheter, however, we would recommend avoiding that if possible due to the above-mentioned reasons (Diamantopoulos et al. 2018). Certainly, a prospective evaluation of a large number of consecutive patients would be needed to evaluate all these technical issues and the clinical outcome. However, that would require multicenter studies including many high-volume centers to overcome the rarity of these scenarios.

A viable and less bulky alternative tibio-peroneal re-entry device may be the Enteer® re-entry catheter (Medtronic, Minneapolis, MN, USA), for which a dedicated type for below the knee interventions is available and which may be easier to apply in the distal vessels of the lower leg. However, at the time of research, there were no studies available about its efficiency and success rate. In our experience this low-profile device may be effective in case of only mild calcification (scenarios I and II). A targeted puncture of the proximal bend of the anterior tibial artery as in scenario III or the application in the setting of the re-entry device assisted SAFARI technique seems unlikely to work. Furthermore, its use requires the storage and training with yet another catheter, while the Outback re-entry device is already widely spread and needs only minor alterations in its usual application.

In the presented scenarios the Outback® device was used only as a bail out option after failure of the usual strategies like the retrograde revascularization. With a technical success rate of about 95%, combined antegrade-retrograde revascularizations, as described by Spinosa et al. as classic SAFARI-technique, are highly efficient (Spinosa et al. 2005). In three of the in this study described cases, the retrograde approach did not lead to successful spontaneous re-entry and had to be combined with puncturing a balloon catheter advanced from the distal crural access point using the Outback® catheter coming from the femoral access. This procedure was described as assisted SAFARI-technique by Tai and Lee in 2015, who used a Philips Pioneer Plus® catheter, which works similar to the Outback® device, with the exceptions that the needle is not aligned by fluoroscopy but by integrated intravascular ultrasound and that the device is even more expensive. This technique has been described only in few case reports so far, but seems to be a promising salvage technique (Tai and Lee 2015).

Yet, the limitations of our study are clear, as we present a retrospective analysis with the inclusion of just a small group of patients. Hence, the documentation of minor complications and the clinical outcome is fragmentary and therefore not meaningfully evaluable. It is obvious and well known that the restoration of blood flow is the key to symptom control and to limb salvage, however, only larger prospective studies could be able to clarify, whether the use of the Outback® device and the subsequent crural stent placement in a large proportion of patients leads to similar clinical results as the standard techniques.

## Conclusion

To reach the essential goal of restoring sufficient blood flow to the foot in patients suffering from critical limb ischemia the use of the Outback® catheter for targeted tibio-peroneal re-entry in case of otherwise failed ante- and retrograde recanalization is a useful salvage option with high technical and subsequently procedural success. In combination with a retrograde access as assisted SAFARI-technique the limits of what is interventionally achievable are extended even further.

## Data Availability

The datasets used and analyzed during the current study are available from the corresponding author on reasonable request.

## Abbreviations

CTO: Chronic total occlusion
IQR: Interquartile range
SAFARI: Subintimal Arterial Flossing with Antegrade-Retrograde Intervention
SIR: Society of Interventional Radiology
SVS: Society of Vascular Surgery
